# Evidence for the Involvement of RhoA Signaling in the Ethanol-Induced Increase in Intestinal Epithelial Barrier Permeability

**DOI:** 10.3390/ijms14023946

**Published:** 2013-02-18

**Authors:** Jing Tong, Ying Wang, Bing Chang, Dai Zhang, Bingyuan Wang

**Affiliations:** Department of Gastroenterology, The First Affiliated Hospital of China Medical University, 155 North Nanjing Street, Shenyang 110001, China; E-Mails: realll30@sina.cn (J.T.); wy_doctor@sina.cn (Y.W.); cb000218@sohu.com (B.C.); realll30@sohu.com (D.Z.)

**Keywords:** RhoA, ethanol, IEB, tight junction

## Abstract

In this work, we investigated the potential role of the small G protein RhoA in ethanol-induced tight junction (TJ) protein disassembly and increased intestinal epithelial barrier (IEB) permeability. Our study used Caco-2 cells as an *in vitro* IEB model and RhoA short hairpin RNA (shRNA) interference to establish whether RhoA plays a role in ethanol-induced TJ opening. RhoA shRNA interference partially inhibited epithelial leakage and restored normal transepithelial electrical resistance (TEER) values in the IEB. Moreover, RhoA shRNA interference prevented a shift in occludin distribution from insoluble to soluble fractions. Additionally, RhoA shRNA interference inhibited the ethanol-induced expression of zonula occludens-1 (ZO-1). Finally, RhoA shRNA interference inhibited an ethanol-induced increase in RhoA activity. The contributions of RhoA to an ethanol-induced increase in IEB permeability are associated with TJ disassembly.

## 1. Introduction

The barrier function of intestinal epithelium is provided by tight junctions (TJ), the highly specialized junctional complexes located at the apical end of epithelial cells. TJ form a barrier to the diffusion of allergens, toxins and pathogens from the intestinal lumen into the interstitial tissue. The disruption of tight junctions increases intestinal permeability to injurious factors, resulting in mucosal inflammation. Tight junctions are organized by interactions between a wide variety of proteins localized specifically at the tight junctions [1]. Occludin, claudins and junction adhesion molecules are the major transmembrane proteins that interact with intracellular plaque proteins, such as zonula occludens (ZOs), including ZO-1, ZO-2 and ZO-3, which in turn interact with the actin cytoskeleton to anchor occludin and other transmembrane proteins at the apical end of the lateral membrane. In the intestine, a disruption of tight junctions may lead to increased permeability to allergens, toxins and pathogens, which appears to be a common mechanism involved in the pathogenesis of a number of gastrointestinal diseases, such as inflammatory bowel disease, celiac disease and alcoholic liver disease (ALD). Most recent studies have addressed the possible effect of ethanol on paracellular permeability and the disruption of epithelial tight junctions using a cell culture model of intestinal epithelium, the Caco-2 cell monolayer [2–5].

Several researchers have investigated the effect of alcohol on intestinal barrier function. Ethanol increases oxidative stress and that increased oxidative stress leads to oxidative damage to the intestinal epithelium and to hyperpermeability of the intestinal barrier [6–8]. Ethanol activates myosin light chain kinase3 and induces nitric oxide generation [9–11], altering the actin and microtubule cytoskeletal structures. Therefore, more than one mechanism may synergistically attenuate barrier function.

RhoA signaling is a crucial regulator of junction assembly and function [12]. Rao [13] reported that botulinum C3 toxin, which inactivates Rho, has been shown to disrupt perijunctional actin, resulting in TJ dysfunction in epithelial cells. In addition, mutant RhoA disrupts TJ function [14]. Thus, signaling pathways transduced by the Ras-related small GTPase Rho family members, such as Rho and Rac1, have been implicated in the regulation of the TJ [15,16]. Consistent with these data, a recently discovered natural RhoA antagonist controls TJ sealing [17].

The aforementioned study suggests that a possible mechanism exists that drives the ethanol-induced opening of the intestinal epithelial barrier (IEB). We propose that RhoA plays a key role in the ethanol-induced opening of the IEB. To test this hypothesis, an IEB model system was used to evaluate the relationship among TJ protein, IEB permeability and RhoA activation.

## 2. Results and Discussion

### 2.1. Cell Viability

**Figure 1:**
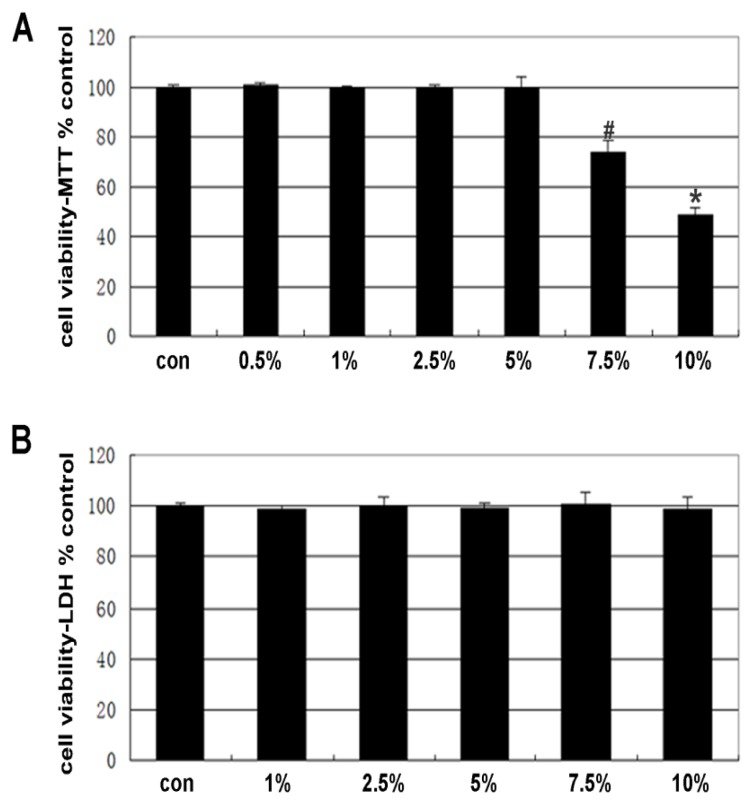
Effect of ethanol on Caco-2 cell viability. Cells were treated with ethanol at different concentrations (0.5%–10%) for 4 h. (**A**) Viability assay performed by the colorimetric MTT method. The formazan product generated was measured at 490 nm and 570 nm. (**B**) The viability was assessed by measuring the released cytosolic enzyme lactate dehydrogenase (LDH) into the medium. Caco-2 cells were incubated in the presence or absence of different concentrations of ethanol for 4 h. Statistically significant differences from the controls are indicated by the colorimetric MTT method: ^#^*p* < 0.01, ^*^p < 0.01. In the LDH assay, no statistically significant difference from the controls was indicated by measuring LDH: *p* > 0.05.

The Caco-2 cells were initially treated for 4 h with different concentrations (1%, 2.5%, 5%, 7.5% and 10%) of ethanol, and cell viability was evaluated by the MTT and LDH assays. The results showed that cell viability, evaluated by the MTT assay, was not altered at ethanol concentrations of less than 5% (Figure 1A). Moreover, at concentrations of up to 10%, ethanol did not increase the release of the cytosolic enzyme lactate dehydrogenase into the medium (Figure 1B). Ethanol concentrations greater than 5% caused cell shedding, but not cell fragmentation. At an ethanol concentration of 10%, the ratio of shedding cells was up to 51.67% ± 3.36%. Therefore, subsequent experiments were carried out using ethanol concentrations ranging from 1% to 5%.

### 2.2. Effect of RhoA on Ethanol-Induced IEB Permeability

**Figure 2:**
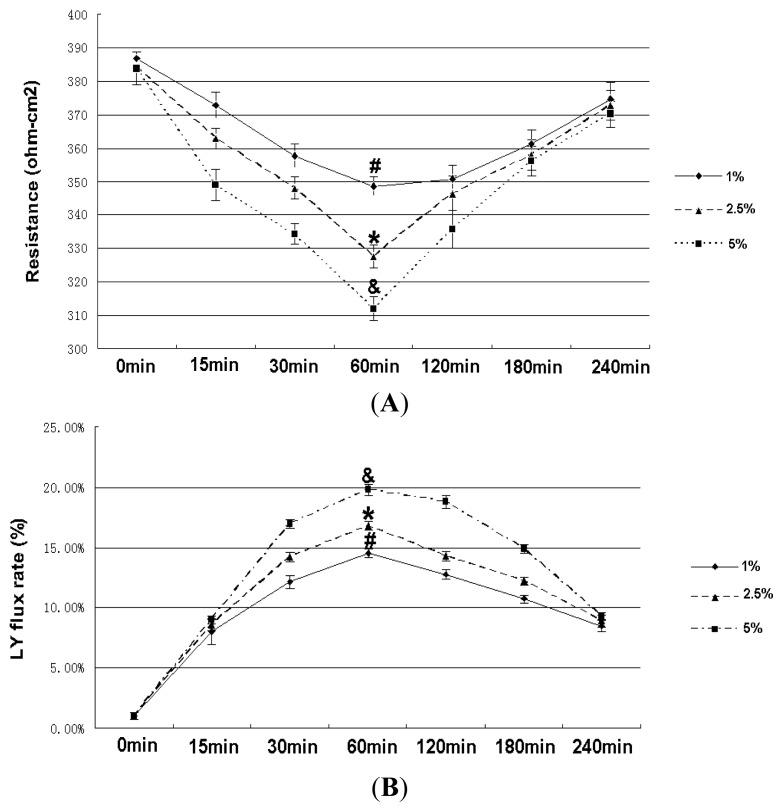
Effect of ethanol and RhoA short hairpin RNA (shRNA) interference on changes in intestinal epithelial barrier (IEB) permeability. (**A**) Effect of ethanol on changes in transepithelial electrical resistance (TEER) of IEB. Caco-2 cells of ethanol groups were treated with 5% ethanol for 0 min, 15 min, 30 min, 60 min, 120 min, 180 min or 240 min. Values are the mean ± SD (*n* = 4 each). One percent ^#^*p* <0.01 *vs.* 0 min group; 2.5% ^*^p < 0.01 *vs.* 0 min group; 5% ^&^*p* < 0.01 *vs.* 0 min group. (**B**) Effect of ethanol on changes in LY flux of the IEB. Caco-2 cells of ethanol groups were treated with 5% ethanol for 0 min, 15 min, 30 min, 60 min, 120 min, 180 min or 240 min. Values are the mean ± SD (*n* = 4 each). One percent ^#^*p* < 0.01 *vs.* 0 min group; 2.5% ^*^p < 0.01 *vs.* 0 min group; 5% ^&^*p* < 0.01 *vs.* 0 min group.

**Figure 3:**
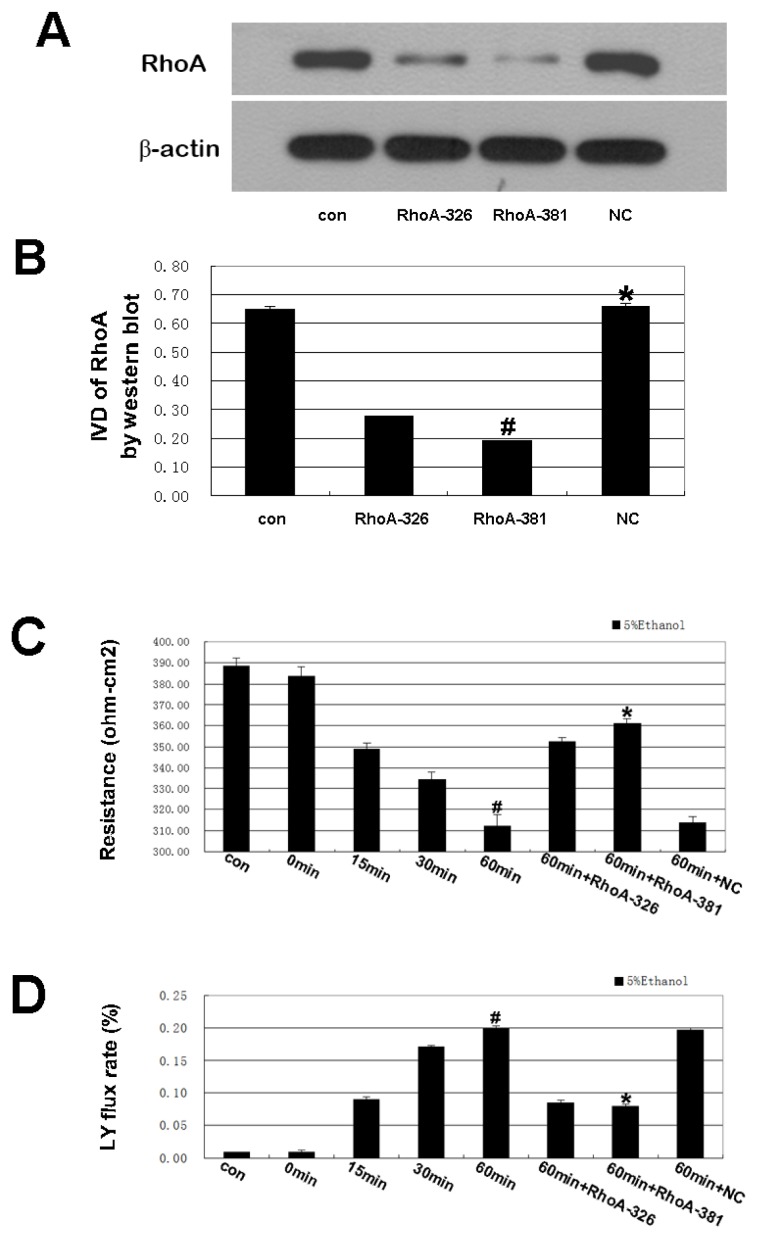
(**A**,**B**) RhoA protein levels measured by Western blot analysis after shRNAs transfection. The blots shown are representative of three independent experiments. The results showed a significantly downregulated protein level of RhoA by two shRNA compared to the control group. Negative control (NC) shRNA group showed no statistically significant decrease expression of RhoA compared to the control group. Values are means ± SD (*n* = 4 each). ^#^*p* < 0.01 *vs.* control group; ^*^p > 0.05 *vs.* control group. (**C**) Effect of RhoA shRNA interference on changes in the TEER of IEB. Caco-2 cells were pretreated with RhoA shRNA interference and then treated with 5% ethanol for 60 min. Values are the mean ± SD (*n* = 4 each). ^#^*p* < 0.01 *vs.* 0 min group. ^*^p < 0.01 *vs.* 60 min group. (**D**) Effect of RhoA shRNA interference on changes in luminal yellow (LY) flux of the IEB. Caco-2 cells were pretreated with RhoA shRNA interference and then treated with 5% ethanol for 60 min. Values are the mean ± SD (*n* = 4 each). ^#^*p* < 0.01 *vs.* 0 min group. ^*^p < 0.01 *vs.* 60 min group.

As shown in Figure 2A, ethanol induced a significant time-dependent decrease in TEER, with the lowest value obtained after 60 min of ethanol treatment. A time-dependent increase in fluorescent yellow flux resulting from the ethanol treatment was observed, with maximum flux occurring at 60 min (Figure 2C). The decreased TEER value was associated with an increase in the fluorescent yellow flux rate. RhoA shRNA partially inhibited ethanol-induced epithelial leakage and restored the normal TEER values and fluorescent yellow flux rate of the IEB (Figure 2B,D). We achieved significant reductions of RhoA protein upon shRNA transfection (Figure 3), and the result showed the RhoA-381 group had higher transfection efficiency. The most effective shRNA was selected for the subsequent experiments. The experiments using luminal yellow (LY) flux and TEER assays were performed mainly to evaluate IEB permeability [18,19]. Our current results indicate that the ethanol-induced increase in IEB permeability is associated with RhoA. Ma *et al.* [3] showed that *in vitro*, ethanol decreased the TEER value in epithelial cells and opened TJ, which is consistent with our findings. Further studies to detect paracellular permeability using the LY flux rate, which is specifically transported through the paracellular space [20], yielded similar TEER results.

### 2.3. RhoA Signal Transduction Activation

**Figure 4:**
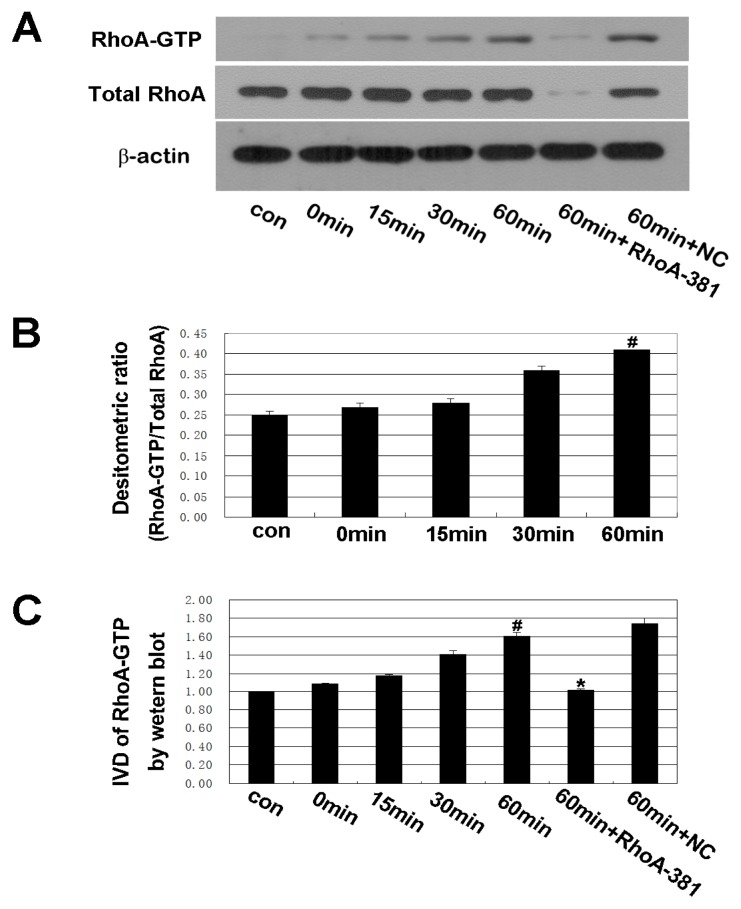
Western blot analysis of the activation of RhoA (RhoA-GTP). Caco-2 cells were treated with 5% ethanol for 0 min, 15 min, 30 min, 60 min, and the ethanol (5%) 60 min + RhoA-381 group was transfected with U6/GFP/Neo-RHOA-homo-381 to block the activity of RhoA and then treated with 5% ethanol for 60 min. (**A**,**B**) The IDV ratio of RhoA-GTP to total RhoA was detected by Western blot analysis. Values are the mean ± SD (*n* = 4 each). ^#^*p* < 0.01 *vs.* 0 min group. (**A**,**C**) The IDV of RhoA-GTP was detected by Western blot analysis. Values are the mean ± SD (*n* = 4 each). ^#^*p* < 0.01 *vs.* 0 min group. ^*^p < 0.01 *vs.* ethanol 60 min group.

As shown in Figure 4, a time-dependent increase in RhoA activity following ethanol administration was observed. The increasing trend was consistent with the ethanol-induced increase in IEB permeability. RhoA shRNA interference inhibited the activation of RhoA at 60 min of treatment. These results suggest that the RhoA pathway may be involved in the ethanol-induced increase in IEB permeability. Rho, a member of a subfamily of small GTPases, is thought to be involved in many cellular functions, including the regulation of actin filament reorganization, cell-shape change and gene expression [21,22]. Extracellular stimuli convert inactive GDP-bound Rho into active GTP-bound Rho. Once activated, Rho interacts with its specific effectors, eliciting a variety of biological functions [23]. Recently, research has demonstrated the important roles of RhoA activation in modulating epithelial permeability [24]. Our studies show that RhoA is required for ethanol-induced changes in IEB permeability.

### 2.4. Inhibition of RhoA Prevented Ethanol-Induced MLC Phosphorylation

**Figure 5:**
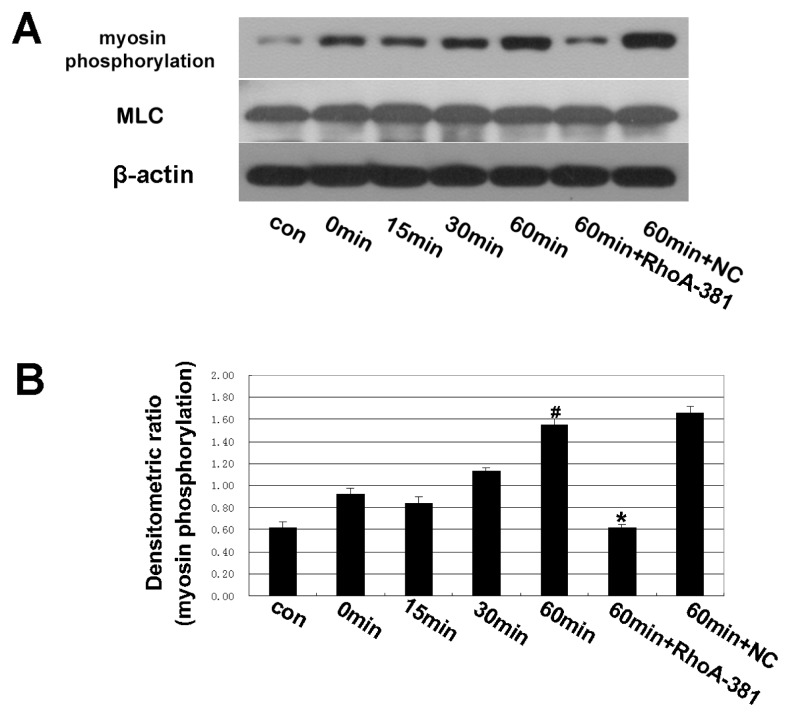
Western blot analysis of myosin phosphorylation and myosin light chains (MLC). Effect of shRNA targeting RhoA on changes in the myosin phosphorylation and MLC expression of Caco-2 cells. Caco-2 cells were pretreated with shRNA targeting RhoA and then treated with 5% ethanol for 60 min. (**A**,**B**) The IDV ratio of myosin phosphorylation and MLC was detected by Western blot analysis.Values are the mean ± SD (*n* = 4 each). ^#^*p* < 0.01 *vs.* 0 min group. ^*^p < 0.01 *vs.* ethanol 60 min group.

As shown in Figure 5, a time-dependent increase in myosin phosphorylation by ethanol administration was observed. RhoA shRNA interference decreased myosin phosphorylation after 60 min. Myosin phosphorylation is a downstream effect of Rho [25–27]. Rho may increase the phosphorylation of myosin light chains (MLC) by decreasing MLC phosphatase activity or by directly phosphorylating MLC at serine 19, its primary phosphorylation site [28–30]. It has also been demonstrated that myosin phosphorylation contributes to TJ disassembly [31–33]. Some investigators have also demonstrated that myosin phosphorylation can disrupt TJ and result in TJ opening [34,35].

### 2.5. RhoA Activity Is Required for Ethanol-Induced Expression and Relocation of Occludin and ZO-1

**Figure 6:**
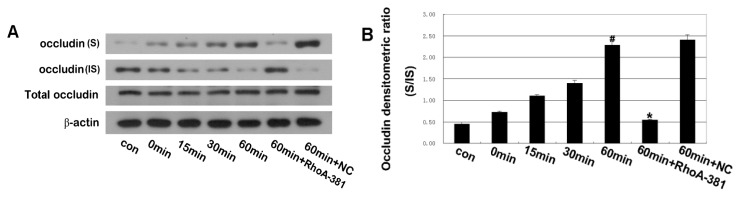

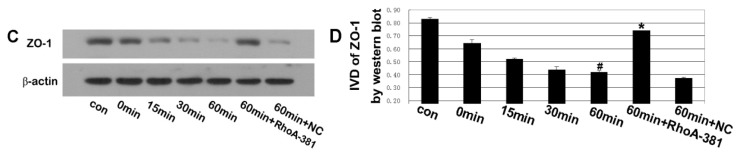
Integrated density value (IDV) ratio of soluble fraction (S) and insoluble fraction (IS) of occludin and IDV of ZO-1 were detected by Western blot analysis. Caco-2 cells were pretreated by shRNA targeting RhoA and then treated with 5% ethanol for 60 min. (**A**,**B**) The IDV ratio of soluble fraction (S) to insoluble fraction (IS) of occludin was detected by Western blot analysis. Values are the mean ± SD (*n* = 4 each). ^#^*p* < 0.01 *vs.* 0 min group; ^*^p < 0.01 *vs.* ethanol 60 min group. (**C**,**D**) Effect of shRNA targeting RhoA on changes in ZO-1 expression of Caco-2 cells. Values are the mean ± SD (*n* = 4 each). ^#^*p* <0.01 *vs.* 0 min group; ^*^p < 0.01 *vs.* ethanol 60 min group.

**Figure 7:**
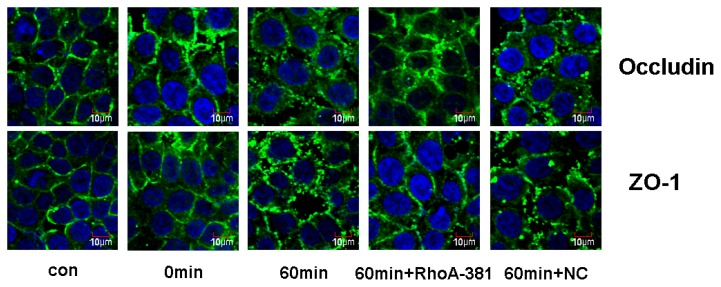
Immunofluorescence localization of occludin and ZO-1 in Caco-2 cells of IEB after ethanol infusion. Caco-2 cells of control, ethanol groups were treated with ethanol (5%) for 0 and 60 min, ethanol (5%) 60 min + RhoA-381 group, ethanol (5%) 60 min + NC group (Caco-2 cells were transfected with U6/GFP/Neo-RHOA-homo-381, pGPU6/GFP/Neo-shNC in the upper chamber of the Transwell and stimulated with ethanol (5%) for 60 min). Occludin and ZO-1 were distributed discontinuously in the boundaries of Caco-2 cells. Scale bars =10 μm.

The integrity of TJ is primarily responsible for epithelial barrier function [36,37]. The strands of TJ are composed of the transmembrane proteins occludin and claudin, polymerizing to form TJs [31]. Occludin is thought to be not only a structural, but also a functional, component of TJs [38]. Occludin, the first identified transmembrane protein of the TJ, is the major regulatory protein of TJs [39–41]. Post-translational modifications of the occludin protein involve phosphorylation events, and multiple phosphorylation sites have been identified on occludin serine and threonine residues [42]. Several proteins, such as Rho kinase, protein kinase C, protein phosphatase 2A and casein kinase 2, may regulate occludin phosphorylation [43,44]. The phosphorylation state of occludin mediates its association with the cell membrane and barrier permeability. Sakakibara *et al.* [45], using phosphoamino acid analyses, showed that phosphorylated occludin protein was selectively recovered in the NP-40-insoluble fraction and appeared in the higher Mr bands, whereas non-phosphorylated occludin protein was predominantly in the NP-40-soluble fraction and appeared in the lower Mr bands. Soluble fragments of occludin are the low molecular weight fragments that are susceptible to non-ionic detergent extraction. Insoluble fragments of occludin represent phosphorylated forms of high molecular weight fragments that combine with actin microfilaments through zonula occludens (ZOs) and that are resistant to non-ionic detergent extraction. As previously reported, a shift in the occludin distribution from insoluble to soluble fractions suggests that the TJs were destroyed [46,47]. Therefore, we prepared the NP-40-soluble and NP-40-insoluble protein fractions for hybridization with an occludin antibody. Our results demonstrated that ethanol induced a shift in the occludin distribution from insoluble to soluble fractions (Figure 6). At the same time, we found that ethanol induced decreased expression of ZO-1 (Figure 6). In our study, ethanol changed the ratio of occludin (S/IS) and expression of ZO-1 in Caco-2 cells, supporting the conclusion that ethanol can affect the expression of TJ proteins, disrupt the TJs and increase the permeability of the intestinal barrier. Moreover, our current data suggest that RhoA shRNA interference prevents the disruption of TJs induced by ethanol treatment (Figure 6). In order to clarify further the mechanism of TJ opening, the distribution of occludin and ZO-1 were assessed by immunofluorescence microscopy (Figure 7). Ethanol induced a re-distribution of occludin from plasmalemma to cytoplasm and induced distribution of ZO-1 discontinuously at the cellular membrane. Our findings reveal that RhoA shRNA interference prevented the ethanol-induced relocation of occludin and ZO-1. Accordingly, it seems possible for a signal transduction pathway to proceed from RhoA to occludin and ZO-1 disassembly, thereby resulting in IEB opening in our presented paper.

RhoA shRNA interference showed only partial reversal of the aforementioned data by ethanol treatment in our study. This result suggests that other signal molecule mechanisms may be involved in ethanol-induced IEB opening.

## 3. Experimental Section

### 3.1. Culture of Caco-2 Cells and Establishment of an *In Vitro* IEB Model

Caco-2 cells were acquired from American Type Culture Collection (ATCC) and cultured in Dulbecco’s Modified Eagles Medium (DMEM, Hyclone, Logan, UT, USA) supplemented with 4.5 mg/mL glucose, 50 U/mL penicillin, 50 U/mL streptomycin, 4 mM glutamine and 10% fetal calf serum (FCS, Hyclone, Logan, UT, USA) in a humidified atmosphere (95% air, 5% CO_2_) at 37 °C. To establish an *in vitro* intestinal epithelial barrier model, Caco-2 cells were plated on Transwell filters (Corning, New York, NY, USA) and monitored regularly by visualization with an inverted microscope and by epithelial resistance measurements.

### 3.2. Viability Assay

The viability assay was performed as described previously [48]. Briefly, Caco-2 cells were grown in 96-well plates, as described above. After incubation with ethanol (1%, 2.5%, 5%, 7.5%, 10%) for 4 h, the cells were incubated with 100 mL of 3-(4,5-dimethylthiazol-2-yl)-2,5-diphenyl tetrazolium bromide (MTT, Sigma-Aldrich, Taufkirchen, Germany) at a concentration of 5 mg/mL MTT diluted in PBS for 1 h at 37 °C. The culture media was removed, and 150 μL of DMSO was added to 96-well plates. The absorbance of the resulting colored solution was measured at 570 nm with a microplate reader (Wallac Victor 2, Perkin-Elmer, Life Sciences, Boston, MA, USA). In this assay, yellow MTT solution is converted into a blue formazan dye within mitochondria and deposited intracellularly. The amount of blue staining is quantitatively detectable by spectrometry and is a measure of cell viability.

At the same time, cell viability was assessed by measuring the release of the cytosolic enzyme. The electrical resistance of Caco-2 cell monolayers cultured on Transwell filters was measured using a Millicell-ERS instrument (Millipore, Bedford, MA, USA). Electrical resistance was expressed in units of Ω cm^2^ using the surface area of the Transwell insert.

Fluorescent yellow (Sigma-Aldrich) at 40 μg/mL in serum-free Dulbecco’s modified Eagle’s medium (DMEM) was added to the upper chamber of the Transwell system. After ethanol was administered for the times indicated, the media from the lower chamber was collected. Absorbance was detected by fluorescence spectrophotometer (excitation wavelength 427 nm, emission wavelength 536 nm), and the fluorescent yellow concentration was calculated according to the standard curve. The fluorescent yellow flux rate (%) = the fluorescent yellow concentration in the lower chamber/the fluorescent yellow concentration in the upper chamber. Caco-2 cells were incubated in different concentrations of ethanol for 4 h. LDH measurement was carried out in 250 μL aliquots using the LDH kit from Doles reagents.

### 3.3. Measurement of Transepithelial Electrical Resistance (TEER) and Fluorescent Yellow Flux Rate Measurement

The electrical resistance of Caco-2 cell monolayers cultured on Transwell filters was measured using a Millicell-ERS instrument (Millipore, Bedford, MA, USA). Electrical resistance was expressed in units of Ω cm^2^ using the surface area of the Transwell insert.

Fluorescent yellow (Sigma-Aldrich, Milwaukee, WI, USA) at 40 μg/mL in serum-free Dulbecco’s modified Eagle’s medium (DMEM) was added to the upper chamber of the Transwell system. After ethanol was administered for the times indicated, the media from the lower chamber was collected. Absorbance was detected by fluorescence spectrophotometer (excitation wavelength 427 nm, emission wavelength 536 nm) and the fluorescent yellow concentration was calculated according to the standard curve. The fluorescent yellow flux rate (%) = the fluorescent yellow concentration in the lower chamber/the fluorescent yellow concentration in the upper chamber.

### 3.4. RNA Interference

To block the activity of RhoA, Caco-2 cells were transfected with U6/GFP/Neo- RHOA-homo-326 and U6/GFP/Neo-RHOA-homo-381 (U6/GFP/Neo-RHOA shRNA expression plasmid, GenePharma, Shanghai, China) using Lipofectamine 2000 (Invitrogen, Carlsbad, CA, USA), according to the manufacturer’s protocol. Negative control sequence (pGPU6/GFP/Neo-shNC) was purchased from GenePharma and used as a control. ShRNA interference efficiency was evaluated by Western blot analysis of the target protein (RhoA) expression. The shRNA sequences were shown in Table 1.

### 3.5. Treatment and Experimental Groups

Caco-2 cells were plated onto the underside of the Transwell insert with suitable culture medium. When the Caco-2 cells were 80% confluent, they were seeded in the upper chamber of the Transwell insert. The cells became confluent after 21–23 days of culture. Ethanol (5%) was added to the upper chamber. The cultured cells were then rinsed with cold phosphate buffered saline (PBS). Subsequently, the Caco-2 cells in the upper chamber were collected by gently scraping with a cell scraper, and the cells were then stored at −80 °C. There were eight groups in our study, with *n* = 4 per group: control group (Caco-2 cells monolayer), ethanol (5%) 0 min group, ethanol (5%) 15 min group, ethanol (5%) 30 min group, ethanol (5%) 60 min group, ethanol (5%) 60 min +RhoA-326 group, ethanol (5%) 60 min + RhoA-381 group, ethanol (5%) 60 min + NC group (respectively, Caco-2 cells were transfected with U6/GFP/Neo-RHOA-homo-326, U6/GFP/Neo-RHOA-homo-381, pGPU6/GFP/Neo-shNC in the upper chamber of the Transwell and stimulated with ethanol (5%) for 60 min).

### 3.6. Cell Fractionation and Western Blot Analysis

The Caco-2 cells were washed three times with Dulbecco’s phosphate-buffered saline (D-PBS) containing 0.1 mM ethylenediamine tetraacetic acid (EDTA) without calcium and magnesium, and the Caco-2 cells were then homogenized in 1 mL of lysis buffer A (2 mM EDTA, 10 mM ethylene glycol tetraacetic acid (EGTA), 0.4% NaF, 20 mM Tris-HCL, protease inhibitor cocktail, phosphatase inhibitor 1% Triton X-100, pH 7.5) at 4 °C. Samples were centrifuged at 14,000× *g* for 30 min, and the supernatant was transferred to a separate tube and collected as the soluble fraction (S). Buffer A (150 μL) with 1% sodium dodecyl sulfate (SDS) at 4 °C was then added to the pellet. The pellet was disrupted with an ultrasonic crusher. The samples were then centrifuged at 14,000× *g* for 30 min at 4 °C, as described above. The supernatant was collected as the insoluble fraction (IS). Equal amounts of proteins (40–50 μg) were separated by SDS-polyacrylamide gel electrophoresis (SDS-PAGE) and processed for immunoblotting with antibodies for MLC, myosin phosphorylation, occludin and ZO-1 (diluted 1:1,000, Santa Cruz Biotechnology, San Francisco, CA, USA). All the protein bands were scanned using ChemiImager 5500 V2.03 software (AlPha InnCh, San Leandro, CA, USA, 2002), and the integrated density values (IDV) were calculated by a computerized image analysis system (Fluor Chen 2.0, Bio-Rad, Hercules, CA, USA) and normalized to that of β-actin.

### 3.7. RhoA Activation Assay

Briefly, the cells were lysed as described above, and the supernatants were collected for protein quantification. Equal amounts of protein were incubated with GST-Rhotekin (Milipore, Billerica, MA, USA) at 4 °C for 1 h with gentle agitation. Affinity-precipitated RhoA proteins were resolved by SDS-PAGE and detected by Western blot analysis. Aliquots of lysates were electrophoresed and blotted in parallel with anti-RhoA (diluted 1:500; Santa Cruz Biotechnology) to determine total RhoA levels (“total RhoA”).

### 3.8. Immunofluorescence

The Caco-2 cell monolayers grown on glass coverslips were fixed with 4% paraformaldehyde and permeabilized with 0.5% Triton X-100. After blocking with 2% BSA in PBS, the cells were incubated with rabbit anti-occludin (diluted 1:50; Santa Cruz Biotechnology) and rabbit anti-ZO-1 (diluted 1:50; Santa Cruz Biotechnology) to visualize the distribution of occludin and ZO-1. The glass slides were analyzed using immunofluorescence microscopy (Olympus, Tokyo, Japan).

### 3.9. Statistical Analysis

Experiments were performed at least three times. Data are presented as the mean ± standard deviation (SD). Comparisons between more than two groups were performed using a one-way analysis of variance (ANOVA), followed by Dunnett’s post-test.

## 4. Conclusions

In summary, our results suggest that RhoA signaling in Caco-2 cells of the IEB is required for the ethanol-induced increase in IEB permeability and that these effects are related to both the RhoA-mediated phosphorylation of MLC and TJ protein disruption.

## Figures and Tables

**Table 1:** shRNA sequences.

Group	shRNA sequences
U6/GFP/Neo-RHOA-homo-326	S5′-CACCGAAAGACATGCTTGCTCATAGTTCAAGAGACTATGAGCAAGCATGTCTTTCTTTTTTG-3′ A5′-GATCCAAAAAAGAAAGACATGCTTGCTCATAGTCTCTTGAACTATGAGCAAGCATGTCTTTC-3′
U6/GFP/Neo-RHOA-homo-381	S5′-CACCGCCCACAGTGTTTGAGAACTATTCAAGAGATAGTTCTCAAACACTGTGGGCTTTTTTG-3′ A5′-GATCCAAAAAAGCCCACAGTGTTTGAGAACTATCTCTTGAATAGTTCTCAAACACTGTGGGC-3′
pGPU6/GFP/Neo-shNC	S5′-CACCGTTCTCCGAACGTGTCACGTCAAGAGATTACGTGACACGTTCGGAGAATTTTTTG-3′ A5′-GATCCAAAAAATTCTCCGAACGTGTCACGTAATCTCTTGACGTGACACGTTCGGAGAAC-3′
